# The Function and Mechanism of Long Noncoding RNAs in Adipogenic Differentiation

**DOI:** 10.3390/genes15070875

**Published:** 2024-07-03

**Authors:** Junhao Chen, Yi Pan, Yunhui Lu, Xue Fang, Tianyi Ma, Xi Chen, Yanhong Wang, Xingtang Fang, Chunlei Zhang, Chengchuang Song

**Affiliations:** Institute of Cellular and Molecular Biology, School of Life Science, Jiangsu Normal University, Xuzhou 221116, China; 1569030404chen@gmail.com (J.C.); yi_pan2002@163.com (Y.P.); lyh211017@163.com (Y.L.); 18752763165@163.com (X.F.); maty_mmm@163.com (T.M.); cxvirus@126.com (X.C.); 7096871@163.com (Y.W.); 6019940077@jsnu.edu.cn (X.F.)

**Keywords:** long non-coding RNA, adipocyte, adipogenic differentiation

## Abstract

Adipocytes are crucial for maintaining energy balance. Adipocyte differentiation involves distinct stages, including the orientation stage, clone amplification stage, clone amplification termination stage, and terminal differentiation stage. Understanding the regulatory mechanisms governing adipogenic differentiation is essential for comprehending the physiological processes and identifying potential biomarkers and therapeutic targets for metabolic diseases, ultimately improving glucose and fat metabolism. Adipogenic differentiation is influenced not only by key factors such as hormones, the peroxisome proliferator-activated receptor (PPAR) family, and the CCATT enhancer-binding protein (C/EBP) family but also by noncoding RNA, including microRNA (miRNA), long noncoding RNA (lncRNA), and circular RNA (circRNA). Among these, lncRNA has been identified as a significant regulator in adipogenic differentiation. Research has demonstrated various ways in which lncRNAs contribute to the molecular mechanisms of adipogenic differentiation. Throughout the adipogenesis process, lncRNAs modulate adipocyte differentiation and development by influencing relevant signaling pathways and transcription factors. This review provides a brief overview of the function and mechanism of lncRNAs in adipogenic differentiation.

## 1. Introduction

Adipose tissue is primarily composed of adipocytes, which store energy in the form of lipids and mobilize it to regulate energy balance [1]. Additionally, adipose tissue serves as a crucial endocrine organ [2], which can secrete a variety of regulatory factors, such as leptin [3], adiponectin [4], and resistin [5]. These factors can activate lipid and glucose metabolism in the body and are anticipated to be therapeutic targets for conditions such as obesity and diabetes [6]. Therefore, a comprehensive understanding of the physiological processes in adipose tissue is of great significance for identifying therapeutic targets for metabolic diseases such as obesity and diabetes.

The differentiation of adipocytes involves distinct stages, including the orientation stage, clonal expansion stage, and terminal differentiation stage [7]. Adipogenesis is a complex biological process regulated by various mechanisms. Key factors in adipogenic differentiation include the transcription factor CCATT enhancer-binding protein (C/EBP) gene family and the peroxisome proliferator-activated receptor-γ (PPAR-γ) [8,9]. In addition to protein-coding genes, noncoding RNAs also play a crucial role in the adipogenic differentiation process. These noncoding RNAs include microRNA (miRNA), long noncoding RNA (lncRNA), and circular RNA (circRNA) [10]. Among them, the role of lncRNAs in regulating adipogenic differentiation has garnered significant attention as research has delved deeper into their function and mechanism. This review primarily focuses on elucidating the role of lncRNAs and their molecular mechanisms in regulating adipogenic differentiation. By understanding the intricate interplay of various factors, including transcription factors and noncoding RNAs, we can gain valuable insights into the complexity of adipogenesis and its implications in metabolic processes.

## 2. Types and Sources of Adipocytes

Adipocytes differentiate from embryonic stem cells (ESCs), and this process involves two cell types: mesenchymal stem cells (MSCs) and preadipocytes [11,12,13]. Traditionally, adipocytes were classified into brown adipocytes and white adipocytes. However, some researchers have identified another type of adipocyte and named it ‘brite’ adipocyte [14,15]. During pregnancy and lactation, the white adipocytes in the mammary glands can undergo conversion into a type of cell known as pink adipocytes. These pink adipocytes possess the ability to produce milk, and it is important to note that this process is reversible [16]. The following sections will introduce these four adipocyte cell types in more detail.

### 2.1. Brown Adipocytes

**Figure 1:**
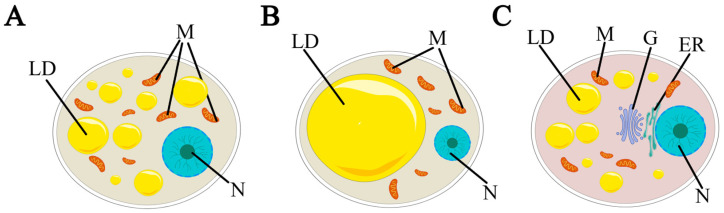
Diagram of different adipocyte cell types. (**A**) Brown adipocyte, (**B**) white adipocyte, (**C**) pink adipocyte. LD, lipid droplet; M, mitochondria; N, nuclear; G, Golgi apparatus; ER, endoplasmic reticulum.

**Figure 2:**
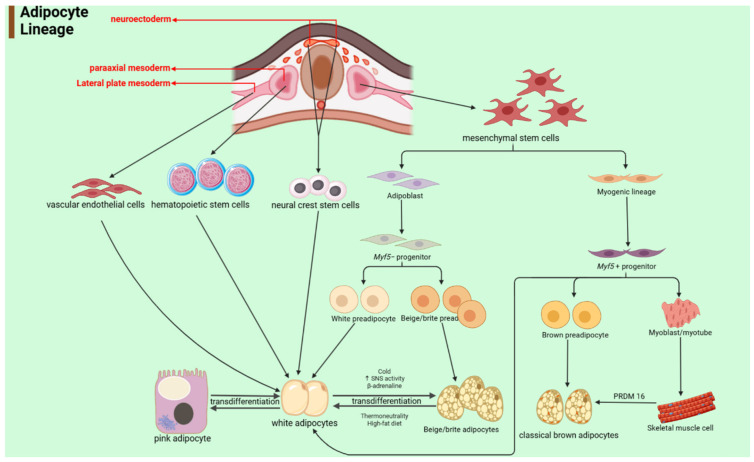
The developmental lineage of different types of adipocytes. (“↑” indicates when there is cold exposure, SNS activation, and elevated levels of β-adrenaline).

Brown adipocytes have multilocular lipid droplets and contain numerous mitochondria, contributing to their brown coloration (Figure 1A) [17]. Uncoupling protein 1 (UCP1) is expressed in brown adipocytes and can be considered a marker of brown adipose tissue [18]. Brown adipocytes can rapidly oxidize their stored fat and circulating substrate, releasing stored energy in the form of heat [19]. Studies in rodents and mammals have demonstrated that the thermogenic activity of brown adipocytes is associated with the prevention of obesity and metabolic diseases such as type 2 diabetes and dyslipidemia [20,21]. In terms of the generation mechanism of brown adipocytes, PRDM16 stands out as a pivotal transcription factor that directs their fate. Under the influence of early B-cell factor-2 (EBF2) and PPARγ, PRDM16 is facilitated in binding to PPARγ, consequently promoting the transition from brown preadipocytes to mature brown adipocytes [22,23,24]. Additionally, brown adipose tissue functions as a secretory organ, releasing a variety of regulatory factors that play a crucial role in vital life processes within the organism [25]. The developmental lineage of brown adipocytes is shown in Figure 2.

### 2.2. White Adipocytes

White adipocytes are dynamic, plastic, and heterogeneous cells. The biological morphology of white adipose adipocytes can be observed in Figure 1B, which can store excess energy in the body in the form of triacylglycerols, thus contributing to obesity. As a component of metabolic homeostasis, it plays a role in a wide range of biological processes, including energy homeostasis, glucose and lipid metabolism, and insulin resistance [26,27,28]. There are two types of white adipose tissue: visceral white adipose tissue and subcutaneous white adipose tissue [29]. White adipocytes can store energy and secrete a variety of cytokines, including adiponectin, leptin, etc., thereby regulating metabolism [27]. Amino acid transporter Asc-1 is a white-adipocyte-specific cell surface protein, which is rarely or not at all expressed in brown adipocytes and can be used to identify and target white adipocytes for disease treatment [30]. In addition, white adipocytes possess the ability to transform into brown-like adipocytes, resulting in intermittent clusters of brown-like adipocytes within white adipose tissue. This intriguing process is referred to as browning [31]. This process is primarily driven by sympathetic stimulation and the interaction of norepinephrine with β3-adrenergic receptors (β3-ARs) present on the plasma membrane of white adipocytes [32]. The key role of browning lies in the heat production process, and β3-AR is the most widely studied receptor that mediates heat production. Activation of the receptor can promote the expression of UCP1, thereby promoting heat production [33]. Its developmental lineage can be seen in Figure 2.

### 2.3. Beige Adipocytes (‘Brite’ Adipocytes)

Beige adipocytes are adipocytes that exhibit similarities to brown fat in morphology and function, but their development more closely resembles that of white adipocytes. They are primarily found within white adipocytes. However, they are a type of adipocyte characterized by a multilocular morphology and functional features, and they can also express UCP1 [23]. The synthesis of beige fat, often referred to as the browning of white fat, has become a significant issue in diabetes and metabolism research [34]. A significant difference from brown adipocytes is that beige adipocytes can express UCP1 and other thermogenic genes when stimulated by activators (beta-adrenoceptor agonists or PPARγ), whereas brown adipocytes express these genes under unstimulated conditions [22]. Furthermore, there are additional distinctions regarding the origin of ‘brite’ adipocytes. In cold environments, ‘brite’ adipocytes may originate from white fat cell precursors in response to environmental stimuli, thereby enhancing heat production. Mature white adipocytes can also be transdifferentiated into beige adipocytes through appropriate contact stimulation [35,36,37]. Interestingly, in addition to endogenous white adipocyte browning caused by changes in hormones and body temperature, there are reports that the browning of white adipocytes may be influenced by food intake, circadian rhythm, and exercise [38].

### 2.4. Pink Adipocytes

A female-specific cell type, the so-called pink adipocyte, has been identified in female mammals [16]. During female pregnancy, these cells are dispersed in the depots of white adipose tissue and trans-differentiate from mature white adipocytes, and they are closely related to milk secretion in the mammary gland [39]. During this period, the white adipose tissue in the mammary gland reversibly differentiates into secretory mammary glands composed of cells rich in cytoplasmic lipids, which are shaped as alveolar structures, and the adipose organs are pink at this time, so the alveolar epithelial cells of the mammary gland are also called pink adipocytes. This process is accompanied by the reduction in adipose tissue and the increase in alveolar structures, and further experimental confirmation is needed to obtain a more detailed understanding of the mechanism of differentiation. After lactation, pink adipocytes transdifferentiate back to white adipocytes or brown adipocytes [16,40,41]. This process holds great significance for breast milk production and infant feeding. A schematic general cytology of pink adipocytes is shown in Figure 1C.

### 2.5. Origin of Adipocytes

Adipocytes are generally thought to originate from the mesoderm, but with the study of adipocyte lineages, the ancestors of white adipocytes do not appear to be that simple. Previous studies used primary and secondary cultures of developing quail neural crest (NC) cells to demonstrate that NC cells can differentiate into adipocytes under the stimulation of specific factors [42]. Reports have indicated that adipocytes can also originate from bone marrow and hematopoietic stem cells (HSCs) [43]. Additionally, thiazolidinediones (TZDs) and high-fat feeding can promote the transformation of bone marrow into adipose tissue and induce differentiation into multilocular adipocytes [44]. Zfp423 is a gene that controls the determination of preadipocytes; immunohistochemistry of Zfp423-driven GFP expression in vivo confirmed a perivascular origin of the preadipocytes within both white and brown adipose tissues [45]. The most common lineage in the origin of adipocytes is myogenic regulatory factor 5 (Myf5). Brown adipocytes and white adipocytes are derived from mesenchymal precursor cell lineages expressing Myf5+ and non-Myf5 (Myf5−), respectively. The pathway for brown adipocyte generation in this context is considered classical [46]. Among them, brown adipose tissue and skeletal muscle share common Myf5+ precursor cells, while white adipocytes do not originate from these cells. This verifies that brown adipose tissue and skeletal muscle have a common origin, but brown adipose tissue and white adipocytes have different origins [24,47,48]. Importantly, mouse skeletal muscle cells transfected with the PRDM16 gene could differentiate into brown adipocytes, and brown adipocytes knocked out of the PRDM16 gene had the appearance and function of skeletal muscle cells. White adipocytes are derived from Myf5- precursor cells; this constitutes the primary pathway for the production of white adipocytes. Otherwise, as mentioned previously, white adipocytes can be converted into UCP1 high-expressing cells (Beige/’brite’ adipocytes) with brown adipocyte characteristics under conditions of β-adrenergic or cold stimulation [14,35,36,37,46]. Exposure to cold activates and recruits brown adipose tissue, resulting in heightened energy expenditure and decreased body fat [49]. Conversely, under thermoneutral conditions, this results in the whitening of brown adipose tissue, accompanied by decreases in mitochondrial content and metabolic activity [50]. The transition between white adipocytes and pink adipocytes has been described above. The developmental lineage of different types of adipocytes is shown in Figure 2.

## 3. Long Noncoding RNAs (lncRNAs) in Adipogenesis

### 3.1. Biogenesis, Characteristics, and Mechanism of lncRNAs

Numerous gene and genomic studies have indicated that up to 90% of eukaryotic genomic DNA can undergo transcription. However, only about 1–2% of these transcriptomes can encode proteins, with the majority transcribed as noncoding RNAs (ncRNAs) [51]. Long noncoding RNAs (lncRNAs) constitute a category of noncoding RNAs characterized by RNA transcripts longer than 200 nucleotides, which lack the ability to be translated into functional proteins [52]. Certainly, it is important to note that not all lncRNAs lack protein-coding potential. Existing studies have demonstrated that a significant number of lncRNAs are indeed associated with ribosomes [53]. The regulatory mechanism of lncRNA is a complex and orchestrated process. In the nucleus, lncRNAs play a role in regulating gene expression at both the transcriptional level and through epigenetic modifications [54]. In the cytoplasm, lncRNAs primarily function as competing endogenous RNAs (ceRNAs), engaging in competition with protein-coding genes by acting as sponges for microRNAs (miRNAs) [55,56]. In this review, our aim is to describe the function and molecular mechanisms of lncRNAs in regulating adipogenic differentiation.

### 3.2. lncRNAs Affect Adipogenic Differentiation by Regulating Transcription Factors

Numerous studies have demonstrated the presence of various transcription factors that play crucial roles in the regulation of adipogenic differentiation. Peroxisome proliferator-activated receptors (PPARs) and the CCATT enhancer-binding protein (C/EBP) family are recognized as critical transcription factors in the process of adipogenesis [57]. Herein, our main focus is summarizing how lncRNAs regulate adipogenic differentiation through the modulation of these transcription factors.

#### 3.2.1. lncRNAs Affect Adipogenic Differentiation by Regulating PPARs

Peroxisome proliferator-activated receptors (PPARs) are representative members of the nuclear receptor (NR) superfamily. PPARs are fatty-acid-activated nuclear receptors consisting of three subtypes: PPARα, PPARβ/δ, and PPARγ [58]. Functionally, PPAR needs to dimerize with the retinoic acid receptor α (RXRα) to form a heterodimeric complex. This complex then binds to specific DNA response elements, activating the expression of target genes [59]. Among the three subtypes, PPARγ is expressed at the highest level in adipose tissue and adipocyte cell lines, whereas in other tissues and cell lines, its expression is relatively low [9,60,61].

Studies have revealed that lncRNA Gm15290 can regulate adipogenesis, and its expression is positively correlated with adipogenic processes. Overexpression of Gm15290 leads to an upregulation in the expression of the transcription factor PPARγ, thereby promoting adipogenic differentiation [62]. lncRNA MIR99AHG functions as a molecular sponge for miR-29p-3p and promotes PPARγ-mediated adipogenic differentiation [63]. lncRNA Plnc1 has the ability to enhance the transcriptional activity of the PPAR-γ2 promoter. This enhancement is attributed to a reduction in the methylation level of the CpG region in the PPAR-γ2 promoter, leading to increased transcription of PPAR-γ2 and ultimately promoting adipogenic differentiation [64]. Overexpression of lncRNA PVT1 has been shown to upregulate the expression of PPARγ, thereby promoting 3T3-L1 adipogenic differentiation [65]. The lncRNA ADNCR functions as a competing endogenous RNA (ceRNA) by sequestering miR-204. This sequestration enhances the target gene SIRT1 expression of miR-204, leading to a reduction in the activity of PPARγ and ultimately inhibiting adipogenic differentiation [66]. The lncRNA H19 can enhance the mRNA expression and protein level of C8orf4, which is downstream of miR-30a. This enhancement leads to a reduced expression level of PPARγ, ultimately resulting in decreased lipid accumulation in human adipose-derived stem cells (hADSCs) and inhibiting adipogenic differentiation [67]. Knockout of the lncRNA KCNQ1OT1 promotes the expression of miR-138 but inhibits the expression of PPARγ. Consequently, this inhibition suppresses adipogenic differentiation in tendon stem cells [68]. From the above, it can be seen that lncRNAs directly or indirectly regulate PPARs to affect adipogenic differentiation.

#### 3.2.2. lncRNAs Affect Adipogenic Differentiation by Regulating C/EBPs

C/EBPs are a family of transcription factors, including C/EBPα, C/EBPβ, C/EBPδ, C/EBPγ, and C/EBPε, and they all contain a highly conserved basic leucine zipper domain at the C-terminus, which is involved in dimerization and DNA binding [69,70,71]. These transcription factors control a series of cell differentiation processes and play a pivotal role in regulating cell proliferation and differentiation by interacting with cyclins [72]. C/EBPγ is the shortest C/EBP protein and lacks a typical activation domain for recruiting transcription [71]. Early studies have shown that C/EBPα plays a major role in adipogenic differentiation [9,73,74,75], while C/EBPβ and C/EBPγ/δ are considered to be involved in directing the process of adipogenic differentiation [75,76,77].

lncRNA Gm15290 [62], lncRNA PVT1 [65], and lncRNA H19 [67,78] mentioned above can regulate not only PPARγ but also C/EBPα. In addition, lncRNA TINCR acts as a ceRNA of miR-31, leading to the upregulation of C/EBPα expression, thereby regulating adipogenic differentiation [79]. The LOXL1-AS1/miR-196a-5p/HMGA2 axis regulates adipogenic differentiation of human bone marrow mesenchymal stem cells (hBMMSCs) by controlling C/EBPβ-mediated PPARγ expression [80]. Firstly, overexpression of LOXL1-AS1 promotes lipogenesis, and LOXL1-AS1 binds to miR-196a-5p and negatively regulates miR-196a-5p expression, so LOXL1-AS1 can act as a molecular sponge for miR-196a-5p. Secondly, Hmga2 is a direct target gene of miR-196a-5p, and Xi et al. [81] showed that Hmga2 can activate C/EBPβ-mediated PPARγ expression to promote lipogenesis. lncRNA ADINR regulates adipogenesis by controlling C/EBPα in cis-transcriptional activity [82]. lncRNA XIST promotes brown preadipocyte differentiation and combats high-fat-diet-induced obesity by directly binding to C/EBPα, as demonstrated through the RNA- binding protein immunoprecipitation (RIP) experiment, indicating that lncRNA XIST is at least partially involved in the differentiation of brown adipocytes by binding to C/EBPα. In addition, in vivo experiments, overexpression of lncRNA XIST can resist HFD-induced weight gain and improve adipose tissue function [83]. From the above, it can be observed that lncRNAs directly or indirectly regulate C/EBPs to impact adipogenic differentiation.

### 3.3. lncRNA Regulates Adipocyte Differentiation by Regulating Different Signaling Pathways

#### 3.3.1. lncRNA Regulates Adipocyte Differentiation through Wnt Signaling Pathway

**Figure 3:**
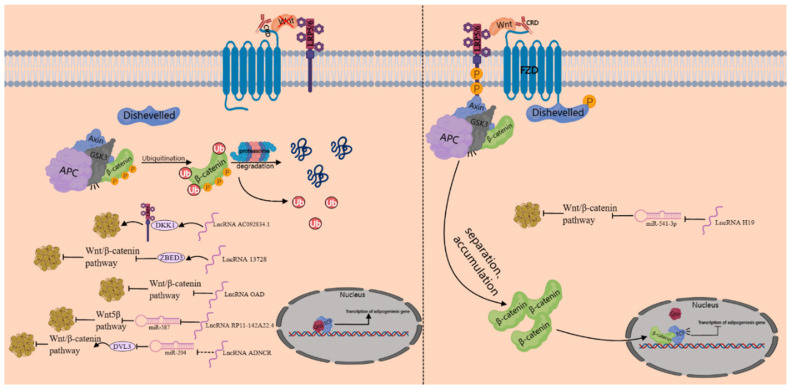
lncRNA regulates adipocyte differentiation through Wnt signaling pathway (“❌” indicates when there is no Wnt signaling).

The Wnt signaling pathway comprises both canonical and noncanonical pathways. The canonical Wnt pathway is also known as the Wnt/β-catenin pathway. The noncanonical Wnt pathway includes the Wnt/Ca^2+^ pathway or the Wnt/planar cell polarity pathway (PCP) [84,85]. Wnt signaling can maintain preadipocytes in an undifferentiated state by inhibiting C/EBPα and PPARγ [86]. lncRNA AC092834.1 inhibits the Wnt-β-catenin pathway by upregulating the expression of DKK1, which competitively binds to LRP5, thereby promoting adipogenic differentiation [87]. lncRNA 13728 may promote the adipogenic differentiation of hADSCs by positively regulating the expression of ZBED3, which subsequently inhibits the Wnt/β-catenin pathway [88]. Melatonin (MT) activates the adiponectin (APN)/Wnt/β-catenin pathway by upregulating lncRNA H19 and inhibiting miR-541-3p, thereby inhibiting the adipogenic differentiation of BMSCs [89]. Overexpressing lncRNA OAD promotes adipogenesis by enhancing mitotic clonal expansion and inhibiting the Wnt/β-catenin signaling pathway [90] (Figure 3). In visceral adipose tissue, an upregulated lncRNA RP11-142A22.4 can regulate preadipocyte differentiation via the miR-587/Wnt5β signaling pathway [91]. lncRNA ADNCR inhibits adipogenic differentiation by serving as a molecular sponge of miR-204 [66]. Interestingly, another study demonstrates that in human-adipose-derived MSCs, miR-204 can inhibit the Wnt/β-catenin signaling pathway by regulating the expression of the target gene DVL3, ultimately promoting adipogenic differentiation [92]. This suggests that there may be some unknown indirect relationship between lncRNA ADNCR and the Wnt/β-catenin signaling pathway.

#### 3.3.2. lncRNA Regulates Adipocyte Differentiation through TGF-β Signaling Pathway

**Figure 4:**
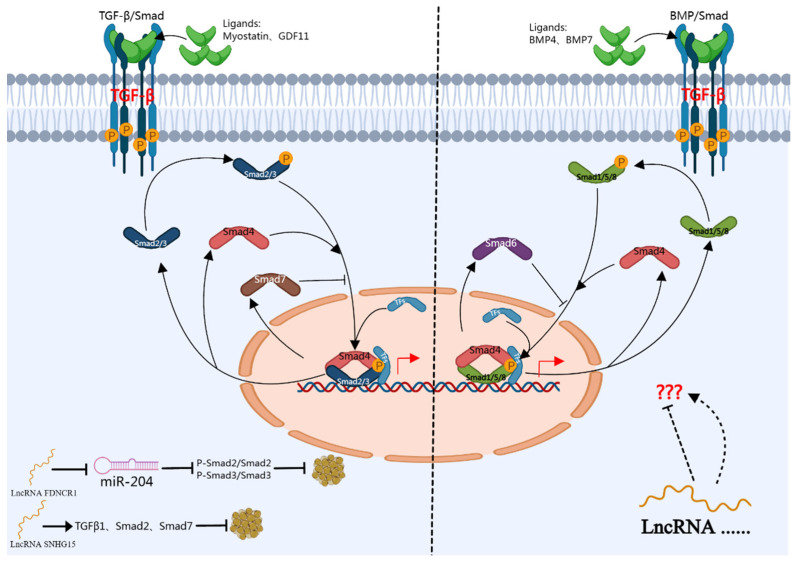
lncRNA regulates adipocyte differentiation through TGF-β/BMPs/Smad signaling pathway. (The right angle arrow indicates the regulation of downstream target gene expression).

The TGF-β superfamily encompasses a diverse array of functions, primarily consisting of TGF-βs, nodal, activin, and BMPs. These members are involved in numerous cellular processes, including adipocyte differentiation [93,94,95] (Figure 4). In porcine adipocytes, TGF-β1-mediated fat-deposition-associated long noncoding RNA1 (FDNCR1), a competitive endogenous RNA (ceRNA) of miR-204, after interference with FDNCR1, mRNA expression and triglyceride levels of aP2, C/EBPα, and PPARγ were significantly increased, but protein levels of Smad2/Smad2 and p-Smad3/Smad3 were significantly decreased. Therefore, FDNCR1 inhibited porcine preadipocyte differentiation through the TGF-β signaling pathway [96]. lncRNA SNHG15 can inhibit adipogenic differentiation of bone marrow mesenchymal stem cells through the TGFβ/Smad signaling pathway under oxidative stress. Under oxidative stress, SNHG15 expression in BMSCs decreased. However, the overexpression of lncRNA-SNGG15 decreased ROS production, increased SOD activity, decreased FABP4 and PPARγ2mRNA expressions, and increased TGFβ1, Smad2, and Smad7 expressions. Thus, upregulation of SNHG15 expression improves the redox balance and inhibits adipogenesis through TGFβ/Smad signaling [97]. In addition, in our previous studies, we derived lncRNAs with various splicing types located near the smad7 gene, including a new splicing variant, named Linc-smad7. Our study also showed that when interfering with Linc-smad7, it can promote the expressions of CEBP α and PPARγ, but when interfering with Linc-smad7 and METTL14 simultaneously, the opposite results were obtained. This indicates that Linc-smad7 regulates lipid synthesis in breast epithelial cells through METTL14 [98]. However, the role of lncRNA in regulating adipogenic differentiation through BMPs/Smad signaling pathway needs further research.

#### 3.3.3. lncRNA Regulates Adipocyte Differentiation through MAPK Signaling Pathway

**Figure 5:**
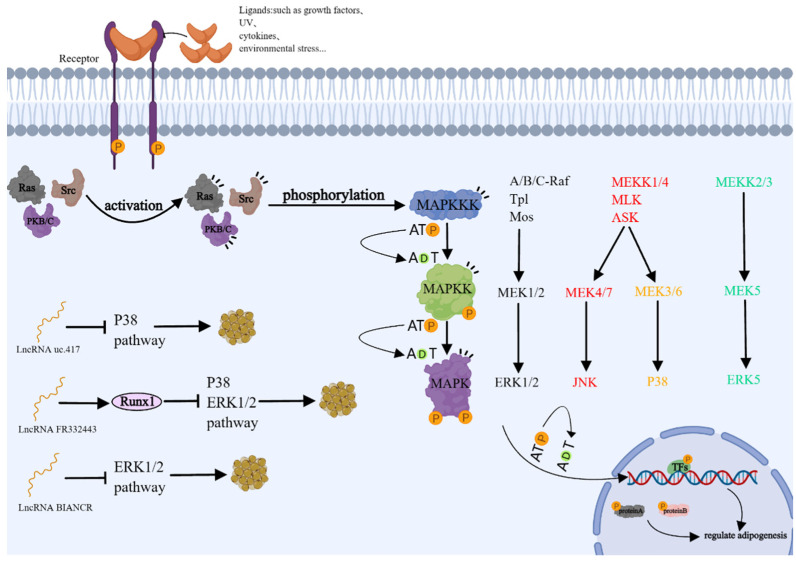
lncRNA regulates adipocyte differentiation through MAPK-related signaling pathway.

The mitogen-activated protein kinase (MAPK) signaling pathways consist of four main subfamilies: the extracellular-signal-regulated kinases (ERK1/2), ERK5, the c-jun N-terminal kinase (JNK1/2/3), and p38-MAPK. These pathways are intracellular signaling cascades involved in numerous crucial cellular processes, including adipogenesis [99,100] (Figure 5). When extracellular stimuli such as growth factors, UV radiation, cytokines, and environmental stress are present, a series of signaling proteins like Ras, tyrosine kinase Src, protein kinase B (PKB), and protein kinase C (PKC) are activated. Subsequently, these signals are transmitted through three main kinases: mitogen-activated protein kinase (MAPK), mitogen-activated protein kinase kinase (MAPKK), and mitogen-activated protein kinase kinase kinase (MAPKKK) [99,101,102].

In brown adipocytes, lncRNA uc.417 suppressed adipogenic differentiation by inhibiting the phosphorylation of p38/MAPK, but it did not affect the total protein level of p38/MAPK [103]. During adipogenic differentiation, lnc-FR332443 and Runx1 are highly enriched in adipose tissue and downregulated. lnc-FR332443 enhances Runx1 expression in mouse adipocytes and suppresses adipocyte differentiation through ERK1/2-MAPK signaling pathways [104]. In intramuscular preadipocytes, knockdown of BIANCR inhibits intramuscular adipogenesis through the ERK1/2 signaling pathway [105]. Together, the lncRNA-mediated ERK1/2-MAPK signaling pathway is an important mechanism for regulating adipogenesis.

#### 3.3.4. lncRNA Regulates Adipocyte Differentiation through PI3K/Akt/mTOR Signaling Pathway

**Figure 6:**
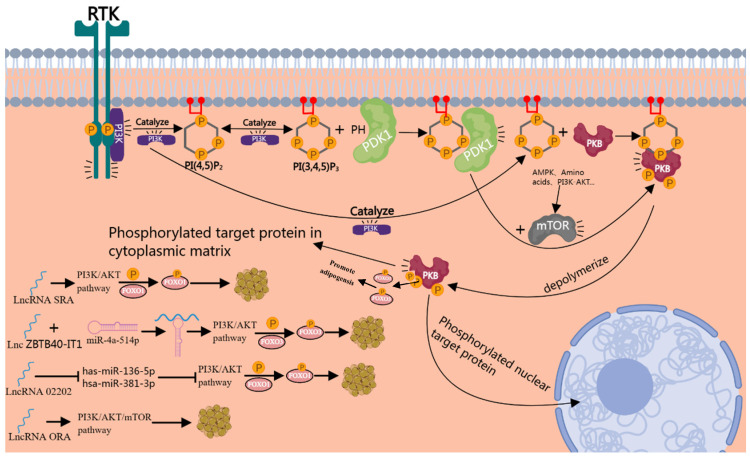
lncRNA regulates adipocyte differentiation through PI3K/Akt/mTOR signaling pathway.

The PI3K/Akt/mTOR pathway is a major intracellular signaling pathway that responds to the availability of nutrients, hormones, and growth factor stimulation [106] (Figure 6). It has been well established to play a significant role in adipogenesis [107,108]. PI3K not only has Ser/Thr kinase activity but also phosphatidylinositol kinase activity, so can catalyze the phosphorylation of phosphatidylinositol 4,5-bisphosphate [PI(4,5)P2], resulting in the formation of phosphatidylinositol 3,4,5-trisphosphate [PI(3,4,5)P3]. The PI(3,4,5)P3 on the plasma membrane can bind to a variety of protein kinases and be activated, and eventually mediate a variety of downstream signal pathways [109]. Protein kinase B (PKB) is a Ser/Thr protein kinase with a molecular weight of about 57kDa. It is a product of retroviral oncogene v-Akt, also known as Akt. Under the phosphorylation of 3-phosphoinositide-dependentproteinkinase1 (PDK1) and PDK2 (usually mTOR), PKB/Akt is completely activated, which in turn phosphorylates a variety of target proteins [109,110,111], such as the forkhead box O family in mammals, including FoxO1, FoxO3, FoxO4, and FoxO6 [112]; when adipogenic proteins are involved, the process of adipogenic differentiation is regulated.

It has been reported that under the stimulation of insulin, the overexpression of lncRNA SRA can not only increase the phosphorylation levels of AKT in Ser-473 and Thr-308 but also increase the phosphorylation level of FOXO1, a downstream target of the AKT signal pathway, indicating that lncRNA SRA may regulate adipogenesis through the AKT signal pathway [113]. Another lncRNA molecule, called ZBTB40-IT1, increases the expression of FOXO3 (a member of the FOXO transcription factor family) through positive binding with miR-4a-514p and finally promotes the adipogenic differentiation of hBMSCs [114]. The members of the FOXO transcription factor family (including FOXO3) are the downstream targets of the PI3K-PKB signal pathway, so we can speculate that lncRNA-ZBTB40-IT1 may activate the PI3K-PKB signal pathway by binding to miR-4a-514p, then phosphorylate FOXO3 and finally promote adipogenic differentiation. Linc-02202 is a novel lncRNA obtained by scholars through bioinformatic analysis [115]. Through analysis, Linc-02202 may act as a ceRNA of has-miR-136-5p/hsa-miR-381-3p to regulate the expressions of PIK3R1 and FOXO,1 respectively. Linc-02202 may also directly affect the transcription of PIK3R1. But, in previous studies, we did not find a role of Linc-02202 in adipogenic differentiation, and thus the above analysis needs further experimental verification. In addition, lncRNA ORA can also regulate adipogenic differentiation through the PI3K/AKT/mTOR signaling pathway [116]. Interfering with lnc-ORA can significantly reduce the expression levels of PPARγ, FASN, and FABP4 and reduce the proportions of p-PI3K/PI3K, p-AKT/AKT, and p-mTOR/mTOR. This indicates that lnc ORA regulates adipocyte differentiation through the PI3K/AKT/mTOR signaling pathway. Overall, the lncRNA-mediated PI3K/AKT/mTOR signaling pathway may be an important way to regulate adipogenesis.

### 3.4. lncRNAs Regulate Adipogenic Differentiation through Epigenetic Mechanisms

Epigenetics refers to heritable changes in gene expression that occur without alterations to the underlying DNA sequence. These changes can be stably transmitted during processes such as development and cell proliferation. Various phenomena contribute to epigenetic regulation, including DNA methylation [117], genomic imprinting [118], maternal effect [119], histone modification [117], chromatin remodeling [120], and RNA modification [121]. lncRNAs can regulate gene expression not only by modulating the chemical modification of histones and DNA but also by controlling the chemical modification of RNA [122]. For instance, lncRNA MIR31HG facilitates the enrichment of AcH3 and H3K4me3 on the fatty acid binding protein 4 (FABP4) gene, consequently enhancing its transcription and promoting adipogenesis [123]. FTO is the first enzyme discovered to catalyze RNA demethylation, and research indicates that FTO’s regulation of adipogenesis is closely linked to RNA m6A modification [124,125]. During the clonal proliferation of 3T3-L1 preadipocyte cells, the loss of FTO function led to enhanced m6A modification levels of CCNA2 and CDK2 mRNAs. These modified sites were recognized by the m6A ‘reading’ protein YTHDF2, resulting in mRNA attenuation and a reduction in protein expression. This process affected RNA stability and ultimately inhibited adipogenesis [126]. In addition, m6A is the most abundant modification in long noncoding RNAs, but it has been rarely reported in adipogenic differentiation. As mentioned earlier, we hypothesized the potential involvement of the m6A-lncRNA axis in adipogenesis. However, further investigation is required to elucidate the underlying molecular mechanisms in depth.

## 4. Prospects

More and more research effort is being directed toward investigating the regulatory mechanisms of adipogenic differentiation. These mechanisms are closely linked to human health, particularly conditions such as obesity and diabetes. In this review, we delve into the role and intricate mechanisms by which lncRNAs regulate various types of adipose differentiation. Recently, with the advancements in high-throughput sequencing technology, tens of thousands of lncRNAs have been identified. However, significant challenges and opportunities still exist in uncovering their functions and molecular mechanisms in adipogenic differentiation. The exact number of lncRNAs playing vital roles in adipocyte differentiation is not yet fully understood, but it is clear that numerous lncRNAs are involved in this process. Additionally, there are likely still novel molecular mechanisms awaiting discovery. The identified lncRNAs offer promising therapeutic targets for the treatment of human diseases, particularly those related to adipose tissue dysfunction such as obesity and diabetes. By understanding their roles and mechanisms, researchers can develop targeted therapies aimed at modulating the expression or function of specific lncRNAs to mitigate disease progression. However, further research efforts are needed to fully elucidate the therapeutic potential of lncRNAs and translate these findings into clinical applications. 

## Data Availability

No new data were created or analyzed in this study. Data sharing is not applicable to this article.
